# Transfer of in vitro-expanded naïve T cells after lymphodepletion enhances antitumor immunity through the induction of polyclonal antitumor effector T cells

**DOI:** 10.1371/journal.pone.0183976

**Published:** 2017-08-30

**Authors:** Tomohiro Tanaka, Satoshi Watanabe, Miho Takahashi, Ko Sato, Yu Saida, Junko Baba, Masashi Arita, Miyuki Sato, Aya Ohtsubo, Satoshi Shoji, Koichiro Nozaki, Kosuke Ichikawa, Rie Kondo, Nobumasa Aoki, Yasuyoshi Ohshima, Takuro Sakagami, Tetsuya Abe, Hiroshi Moro, Toshiyuki Koya, Junta Tanaka, Hiroshi Kagamu, Hirohisa Yoshizawa, Toshiaki Kikuchi

**Affiliations:** 1 Department of Respiratory Medicine and Infectious Diseases, Niigata University Graduate School of Medical and Dental Sciences, Niigata City, Niigata, Japan; 2 Respiratory Medicine, Saitama International Medical Center, Saitama, Japan; 3 Bioscience Medical Research Center, Niigata University Medical and Dental Hospital, Niigata City, Niigata, Japan; CCAC, UNITED STATES

## Abstract

The adoptive transfer of effector T cells combined with lymphodepletion has demonstrated promising antitumor effects in mice and humans, although the availability of tumor-specific T cells is limited. We and others have also demonstrated that the transfer of polyclonal naïve T cells induces tumor-specific effector T cells and enhances antitumor immunity after lymphodepletion. Because tumors have been demonstrated to induce immunosuppressive networks and regulate the function of T cells, obtaining a sufficient number of fully functional naïve T cells that are able to differentiate into tumor-specific effector T cells remains difficult. To establish culture methods to obtain a large number of polyclonal T cells that are capable of differentiating into tumor-specific effector T cells, naïve T cells were activated with anti-CD3 mAbs in vitro. These cells were stimulated with IL-2 and IL-7 for the CD8 subset or with IL-7 and IL-23 for the CD4 subset. Transfer of these hyperexpanded T cells after lymphodepletion showed significant antitumor efficacy, and tumor-specific effector T cells were primed from these expanded T cells in tumor-bearing hosts. Moreover, these ex vivo—expanded T cells maintained T cell receptor diversity and showed long-term persistence of memory against specific tumors. Further analyses revealed that combination therapy consisting of vaccination with dendritic cells that were co-cultured with irradiated whole tumor cells and the transfer of ex vivo—expanded T cells significantly enhanced antitumor immunity. These results indicate that the transfer of ex vivo—expanded polyclonal T cells can be combined with other immunotherapies and augment antitumor effects.

## Introduction

Lymphodepletive cytotoxic regimens, such as chemotherapy and radiotherapy, have demonstrated the ability to augment antitumor immunity. In particular, the antitumor efficacy of tumor-specific effector T cells is clearly enhanced when they are transferred into lymphopenic tumor-bearing hosts [1]. We and others have found that transfer of naïve T cells and effector T cells enhanced antitumor immune responses and inhibited tumor progression [2] [3, 4]. Polyclonal naïve T cells transferred into lymphopenic hosts proliferate rapidly and differentiate into antitumor effector T cells. Previous studies have suggested that lymphodepletion augments antitumor immunity through the depletion of immune-suppressor cells [5, 6]. Recent studies have further shown that lymphodepletion decreases host cell competition for activating cytokines, such as IL-7, IL-15 and IL-21, and increases the availability of these cytokines to transferred T cells [7, 8]. Furthermore, we have previously demonstrated that the percentage of regulatory T cells (Tregs) increases after lymphodepletion [3, 4]. The Tregs that survive lymphodepletion suppress the development of antitumor immunity during recovery from lymphopenia, and the depletion of Tregs following lymphodepletion augments antitumor immune responses.

Although the induction of tumor-specific effector T cells via the transfer of naïve T cells following lymphodepletion seems to be a promising approach to augment antitumor immunity, a large number of naïve T cells must be collected from tumor-bearing hosts. Previous studies have demonstrated that recognition of self-antigens by the T cell receptor (TCR) is important for the proliferation of T cells during lymphopenia-induced homeostatic proliferation [9] [10]. However, TCR functions are impaired in tumor-bearing hosts [11] [12]. Tumor cells induce immunosuppressive mechanisms, such as the induction of regulatory cell populations and the secretion of immunosuppressive soluble factors, and they also inhibit the function of antitumor T cells [13, 14] [15]. Thus, it remains difficult to harvest a sufficient number of fully functional naïve T cells from cancer patients. In the current study, we investigated whether ex vivo—expanded naïve T cells show antitumor efficacy when they are transferred into lymphopenic tumor-bearing hosts. We and others have previously reported that effector T cells purified from tumor-draining lymph nodes (TDLNs) can be efficiently expanded in complete medium (CM) supplemented with specific cytokines following anti-CD3 activation [16] [17]. Moreover, the transfer of these ex vivo—expanded effector T cells eliminated established tumors. In this study, we stimulated naïve T cells from the spleen of normal mice with immobilized-anti CD3 monoclonal antibodies (mAbs). These T cells were further stimulated in CM supplemented with IL-2 and IL-7 for the CD8 subset or with IL-7 and IL-23 for the CD4 subset. The resultant cells were transferred into irradiated lymphopenic tumor-bearing mice. Ex vivo—expanded T cells from naïve mice were differentiated into effector T cells in lymphopenic hosts and inhibited tumor progression. Effector T cells primed from these ex vivo—expanded T cells from naïve mice were long-lived and rejected specific tumor rechallenge. Moreover, the combination of dendritic cell (DC) vaccination and the transfer of ex vivo—expanded T cells had potent antitumor effects. These results indicate that ex vivo expansion of naïve T cells may yield a sufficient number of fully functional T cells from cancer patients to augment antitumor immunity in clinical settings.

## Materials and methods

### Animals

Female C57BL/6N (B6) mice were purchased from CLEA Laboratory (Tokyo, Japan). Ly5.1 congenic B6 mice were obtained from Sankyo Labo Service (Tokyo, Japan). OT-II transgenic mice and Rag2^-/-^ mice were purchased from the Jackson Laboratory (Bar Harbor, ME). The mice were housed in a specific pathogen-free environment and used at an age of 8 to 12 weeks. The experimental protocols were approved by the Niigata University Institutional Animal Care and Use Committee.

### Tumors

The 3-methylcholanthrene (MCA)-induced fibrosarcoma cell lines MCA205 and MCA207, originally derived from B6 mice, were routinely passaged in vivo and used between the fifth and eighth passages [18]. Single-cell suspensions were prepared from solid tumors by digestion with a mixture of 0.1% collagenase, 0.01% DNase, and 2.5 U/ml hyaluronidase (Sigma-Aldrich, St. Louis, MO) for 3 hours at room temperature. The cells were filtered through a 100-μm nylon mesh, washed, and suspended in HBSS for intravenous (i.v.) and subcutaneous (s.c.) inoculations.

### Cell culture

Animals were sacrificed by cervical dislocation and the spleens were excised. Naïve spleen cells were depleted of CD4^+^ or CD8^+^ cells by negative selection with mAb-coated magnetic beads (Invitrogen, Carlsbad, CA). CD8- or CD4-depleted cells were suspended in CM and activated for 2 days at 4 × 10^6^ cells per well in 24-well culture plates coated with anti-CD3 mAb (145-2C11). Activated CD8-depleted cells were suspended at 0.5 × 10^6^ /ml in CM with IL-7 (10 ng/ml; R&D Systems, Minneapolis, MN) and IL-23 (2 ng/ml; R&D Systems, Minneapolis, MN). Activated CD4-depleted cells were suspended at 0.5 × 10^6^ in CM with human recombinant IL-2 (16 U/ml; kindly supplied by Shionogi) and IL-7 (10 ng/ml). For long-term expansion, the cells were re-stimulated with anti-CD3 mAb for 14 hours on days 21 to 23. CM consists of RPMI 1640 supplemented with 10% heat-inactivated fetal bovine serum and antibiotics.

### Adoptive transfer

B6 mice were lymphodepleted by sublethal irradiation with 500 cGy. On the same day, the mice were reconstituted i.v. with T cells that had been expanded in vitro. These mice were then inoculated s.c. with 1 × 10^5^ MCA205 tumor cells along the midline of the abdomen. Tumor sizes were measured in 2 perpendicular dimensions 2 to 3 times per week with digital calipers and recorded as the tumor area (mm^2^).

### Activation of TDLN cells

The generation of activated TDLN cells has been described previously [16]. Briefly, B6 mice were inoculated s.c. with 3 × 10^6^ MCA205 tumor cells on both flanks to stimulate TDLNs. Twelve days later, TDLNs (inguinal) were harvested, and single-cell suspensions were prepared mechanically. These TDLN cells were activated with anti-CD3 mAb immobilized on 24-well plates for 2 days and expanded in CM containing 16 U/ml recombinant human IL-2 for 3 days.

### FACS analysis and in vivo proliferation

FITC-conjugated mAbs against CD11c (HL3), CD25 (PC61) and the Mouse Vβ Screening Panel kit; PE-conjugated mAbs against CD4 (RM4-5), CD8 (53–6.7), CD25 (PC61), CD44 (IM7), CD62L (MEL-14), CD69 (H1.2F3), CD80 (16-10A1), CD86 (GL1), CD95 (Jo2), CCR7 (4B12), CTLA-4 (UC10-4F10-11), H-2k^b^ (AF6-88.5), I-A^b^ (AF6-120.1) and IFN-γ (XMG1.2); PE-Cy7-conjugated mAbs against CD4 (RM4-5), CD8 (53–6.7) and Ly5.1 (A20); and isotype-matched mAbs were purchased from BD Biosciences. PE-anti-Forkhead box P3 (Foxp3, FJK-16s) was purchased from eBioscience. The cell surface phenotypes were determined by direct immunofluorescence staining with conjugated mAbs and analyzed using a FACSCalibur flow cytometer (BD Biosciences, San Jose, CA). Foxp3 staining was performed using the PE-Foxp3 staining set (eBioscience, San Diego, CA). For the in vivo proliferation assay, cultured T cells and spleen T cells from normal mice were labeled with CFSE (Molecular Probes, Eugene, OR). Briefly, T cells were suspended at 1 × 10^7^ cells/ml and incubated with CFSE in HBSS for 10 minutes at 37°C. The labeling was stopped by adding ice-cold HBSS, and the cells were washed twice with HBSS before being transferred into irradiated mice.

### Intracellular IFN-γ staining

Intracellular IFN-γ staining was performed as previously described [19]. Briefly, activated T cells were stimulated with a single-cell suspension of either MCA205 or MCA207 tumor cells prepared from solid tumor tissues at a 1:1 ratio. Controls included stimulation with immobilized anti-CD3 mAbs. Brefeldin A (10 μg/ml, Sigma-Aldrich, St. Louis, MO) was added at 6 hours, and the cells were harvested at 24 hours. The cells were then pretreated with FcR-blocking Abs, followed by staining for 30 minutes with PE-Cy7-conjugated anti-CD4 or anti-CD8 mAbs. Washed cells were fixed with 2% paraformaldehyde for 20 minutes, permeabilized with 0.3% saponin, and incubated for 40 minutes with PE-conjugated IFN-γ at 4°C. Unbound mAbs were removed by two washes with 0.3% saponin in PBS.

### Preparation of DCs

DCs were generated from bone marrow cells according to a previously described procedure [20]. In brief, bone marrow cells obtained from femurs and tibias of naive mice were placed in T-75 flasks for 2 h at 37°C in CM containing 10 ng/ml recombinant murine GM-CSF (kindly supplied by KIRIN). Nonadherent cells were collected by aspirating the medium and transferred into fresh flasks. On day 6, nonadherent cells were harvested by gentle pipetting. Approximately 80% CD11c^+^CD11b^+^ cells and 20% CD11c^−^CD11b^+^ cells were obtained.

### Activation of DCs

To settle DCs on a T-25 flask precoated with goat anti-rat Ig Ab and anti-CD40 Ab (3/23; Bio-Rad, Oxford, UK) as soon as possible, the cells were plated in 4 ml of CM supplemented with GM-CSF (10 ng/ml). Anti-CD40 Ab-coated flasks were prepared as described below. T-25 flasks were coated with 2 ml of goat anti-rat Ig Ab solution (100 μg/ml) overnight at 4°C. The Ab solution was removed, and the flasks were washed twice with PBS. The flasks were further coated with rat anti-murine CD40 Ab (100 μg/ml) for 30 minutes at 37°C in a 5% CO_2_ incubator and used immediately after washing with PBS.

### Statistical analysis

The significance of the differences between groups was analyzed using unpaired two-tailed Student’s *t* test as indicated in the figure legends. When data failed normality test, the Wilcoxon rank-sum test was performed to evaluate statistical significance. Results were presented as mean ± SEM. A *p* value < 0.05 was considered significant. All experiments were repeated at least twice. Statistical analyses were performed using JMP version 9 software (SAS Institute, Cary, NC).

## Results

### Ex vivo expansion of naïve CD4^+^ and CD8^+^ T cells under specific culture conditions

**Fig 1:**
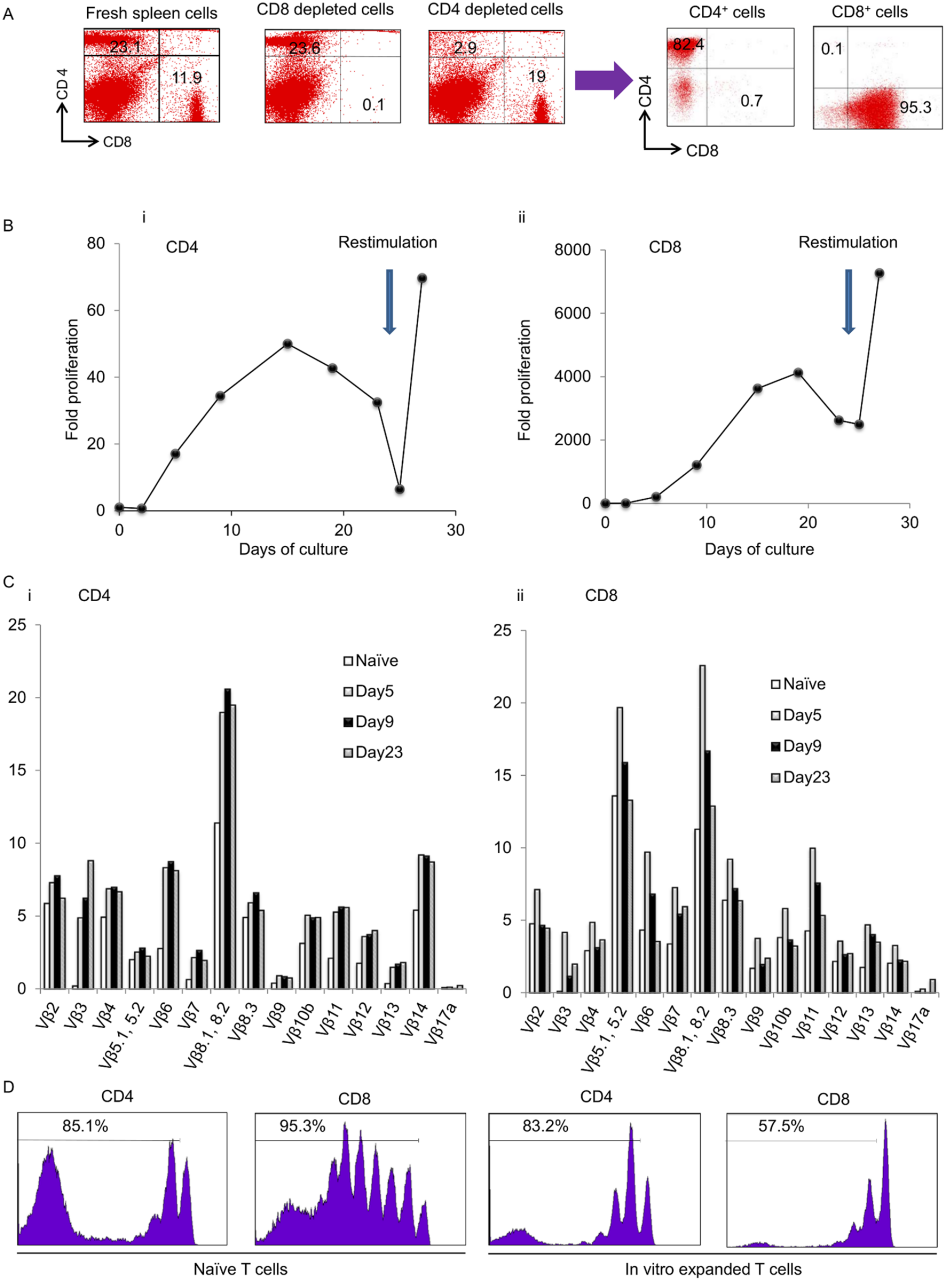
Ex vivo proliferation of CD4^+^ and CD8^+^ T cells. (A) CD4^+^ or CD8^+^ T cells from normal spleen cells were magnetically depleted in vitro. These CD4- and CD8-depleted cells were activated with immobilized CD3 mAbs for 2 days. CD4-depleted cells were subsequently stimulated with IL-2 and IL-7, whereas CD8-depleted cells were stimulated with IL-7 and IL-23. Dot grams showing the percentage of CD4^+^ and CD8^+^ cells before and 9 days after stimulation. (B) CD8-depleted (Bi) and CD4-depleted cells (Bii) were activated with anti-CD3 followed by stimulation with IL-7 plus IL-23 or IL-2 plus IL-7, respectively. On day 23, the resultant cells were re-stimulated with plate-bound anti-CD3 mAbs for 14 hours. The fold proliferation of CD4^+^ and CD8^+^ cells is shown. (C) Normal spleen cells were depleted of CD8^+^ cells or CD4^+^ cells and were activated under specific culture conditions. The percentages of TCR-Vβ subpopulations in CD4^+^ (Ci) and CD8^+^ (Cii) T cells after ex vivo expansion are demonstrated. Representative results from three independent experiments are shown. (D) Ex vivo—expanded CD4^+^ and CD8^+^ T cells from congenic Ly5.1^+^ spleen cells were labeled with CFSE and transferred i.v. into sublethally irradiated Ly5.2^+^ mice. These mice were inoculated s.c. with MCA205 tumor cells in the right flank to stimulate the TDLNs. Twelve days later, TDLNs (inguinal) were harvested and analyzed for the CFSE staining intensity within the Ly5.1^+^ subset. Data shown are from a single experiment representative of three independent experiments.

We have repeatedly demonstrated that effector T cells are primed in TDLNs and that the transfer of these cells produces antitumor effects after activation with CD3/IL-2 ± IL-7 [16, 21] [22]. Further experiments revealed that CD4^+^ and CD8^+^ effector T cells could be efficiently expanded under specific culture conditions [17]. CD4^+^ effector T cells that were primed in TDLNs and stimulated with IL-7 and IL-23 inhibited tumor progression more strongly than those stimulated with IL-2 and IL-7 when they were transferred into lymphopenic tumor-bearing mice. To determine whether naïve T cells were also efficiently expanded in vitro, we first depleted CD4^+^ or CD8^+^ cells from naïve spleen cells (Fig 1A). CD4-depleted cells and CD8-depleted cells were then activated with plate-bound anti-CD3 mAb for 2 days. CD4-depleted cells were subsequently cultured in CM supplemented with IL-2 and IL-7. By contrast, CD8-depleted cells were expanded in the presence of IL-7 and IL-23. On day 9 of culture, FACS analyses showed that 82.4% of the CD8-depleted cells were CD4^+^ cells and 95.3% of the CD4-depleted cells were CD8^+^ cells. Fig 1B shows the fold proliferation of CD4^+^ and CD8^+^ cells. Similar to previous experiments, re-stimulation with immobilized anti-CD3 mAb for 14 hours on day 23 resulted in further expansion of CD4^+^ and CD8^+^ cells [17].

Next, we investigated whether these ex vivo—expanded naïve T cells could maintain the diversity of the T cell repertoire. FACS analyses revealed a broad overall TCR Vβ usage in CD4^+^ and CD8^+^ T cells after days of culture (Fig 1C).

We previously observed that the transfer of naïve T cells into lymphopenic tumor-bearing mice resulted in potent antitumor effects [3, 4]. Naïve T cells proliferated peripherally in lymphopenic hosts and acquired antitumor effector functions. To evaluate the proliferation of ex vivo—expanded naïve T cells in lymphopenic mice, naïve CD4^+^ and CD8^+^ T cells from congenic Ly5.1 mice were separately stimulated for 9 days as described above. These cells or freshly harvested naïve spleen cells (40 × 10^6^) from Ly5.1 mice were stained with CFSE and transferred i.v. into sublethally (500 cGy) irradiated Ly5.2 mice. Next, the Ly5.2 mice were inoculated s.c. with MCA205 cells (3 × 10^6^) to stimulate TDLNs. Twelve days later, inguinal TDLNs were harvested. The proliferation of Ly5.1^+^ donor CD4^+^ and CD8^+^ T cells was evaluated via CFSE dilution. As shown in Fig 1D, ex vivo—expanded CD4^+^ T cells (83.2%) had proliferated together with naïve CD4^+^ T cells (85.1%). By contrast, ex vivo—expanded CD8^+^ T cells (57.5%) had divided less than naïve CD8^+^ T cells (95.3%).

### Ex vivo—Expanded CD4^+^ T cells show antitumor effects in lymphopenic mice

**Fig 2:**
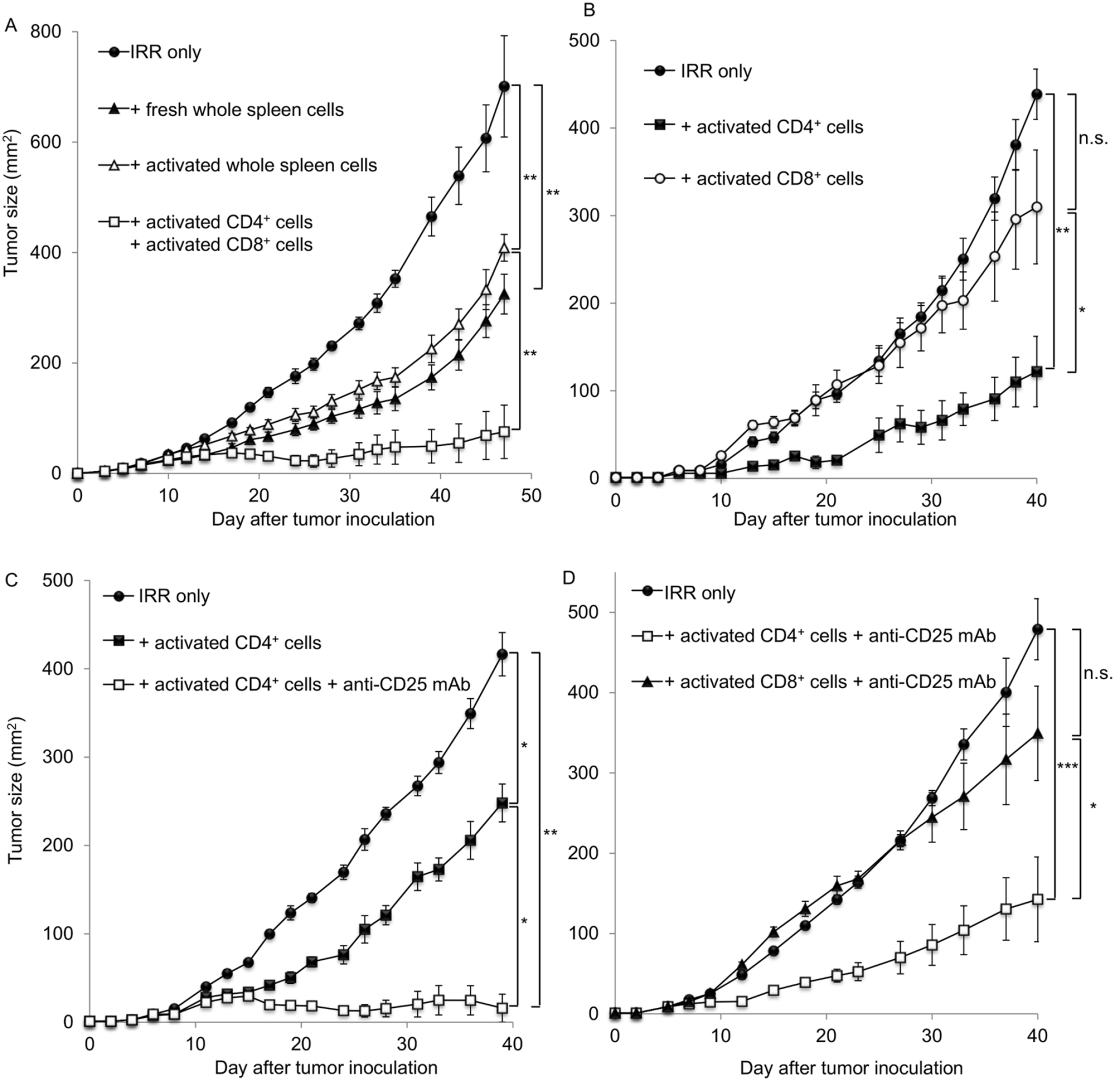
Transfer of ex vivo—Expanded CD4^+^ T cells enhanced antitumor immunity. (A) Irradiated lymphopenic mice were reconstituted with either freshly harvested naïve spleen cells, whole spleen cells that were stimulated with CD3/IL-2+IL-7 for 9 days, or CD4^+^ T cells that were stimulated with anti-CD3/IL-7+IL-23 and CD8^+^ T cells that were stimulated with anti-CD3/IL-2+IL-7 for 9 days. These mice were inoculated s.c. with MCA205 tumor cells along the midline of the abdomen. The resultant skin tumors were measured in two perpendicular directions two to three times per week, and the tumor areas (mm^2^) were recorded. (B) Irradiated mice were transferred i.v. with ex vivo—expanded CD4^+^ or CD8^+^ T cells and were inoculated s.c. with MCA205 tumor cells. (C) Irradiated mice were transfused i.v. with ex vivo—expanded CD4^+^ T cells. These mice were injected i.p. with anti-CD25 mAbs following the inoculation of MCA205 tumor cells. (D) Irradiated mice were reconstituted with either ex vivo—expanded CD4^+^ or CD8^+^ T cells. These mice were inoculated s.c. with MCA205 tumor cells and treated with anti-CD25 mAbs to deplete Tregs. Data are shown as mean ± SEM of 5 mice per group and are from one experiment representative of two to three independent experiments. *p < 0.05, **p < 0.01, ***p < 0.001 (two sided Student’s *t* test).

We next investigated whether the transfer of ex vivo—expanded T cells into irradiated lymphopenic mice could augment antitumor immunity. Sublethally irradiated mice were injected i.v. with either ex vivo—expanded T cells (30 × 10^6^) as described above, whole spleen cells that were stimulated in vitro with anti-CD3/IL-2+IL-7 for 9 days or freshly harvested naïve spleen cells. These mice were inoculated s.c. with MCA205 tumor cells (1 × 10^5^) along the midline of the abdomen on the same day. Consistent with previous studies, the transfer of fresh spleen cells after irradiation delayed skin tumor growth (Fig 2A). Although the transfer of activated whole spleen cells delayed skin tumor growth, the transfer of ex vivo—expanded CD4^+^ T cells and CD8^+^ T cells strongly inhibited tumor progression (Fig 2A). To determine whether the transfer of CD4^+^ and CD8^+^ T cells was responsible for the augmentation of antitumor immunity in this model system, irradiated lymphopenic mice were reconstituted with ex vivo—expanded CD4^+^ T cells or CD8^+^ T cells and then challenged with MCA205 tumor cells. As shown in Fig 2B, the transfer of CD8^+^ T cells was associated with minimal antitumor efficacy. By contrast, significant retardation of skin tumor growth was observed in mice reconstituted with CD4^+^ T cells.

In previous studies, we demonstrated an increase in the percentage of Tregs in irradiated lymphopenic mice [3]. Tregs that survive sublethal irradiation suppress the development of antitumor immunity during recovery from lymphopenia, whereas depletion of these irradiation-resistant Tregs significantly enhances the antitumor effects of the transfer of naïve T cells following irradiation. To investigate whether the depletion of radio-resistant Tregs enhanced the antitumor efficacy of the ex vivo transfer of expanded CD4^+^ T cells after irradiation, irradiated lymphopenic mice were transferred i.v. with ex vivo—expanded CD4^+^ T cells and then inoculated s.c. with MCA 205 tumor cells. These mice were injected intraperitoneally (i.p.) with anti-CD25 mAb (PC61) to deplete Tregs on the same day. As demonstrated in Fig 2C, the depletion of Tregs following irradiation and the transfer of ex vivo—expanded CD4^+^ T cells significantly inhibited skin tumor growth. Although the transfer of ex vivo—expanded CD8^+^ T cells into lymphopenic mice showed minimal antitumor effects, we evaluated whether the depletion of Tregs enhanced the antitumor effects of ex vivo—expanded CD8^+^ T cells. In contrast to the transfer of ex vivo—expanded CD4^+^ T cells, the transfer of ex vivo—expanded CD8^+^ T cells followed by the depletion of Tregs did not show strong antitumor effects (Fig 2D).

### Effector CD4^+^ T cells are primed from ex vivo—Expanded CD4^+^ T cells and show maintenance of TCR diversity

**Fig 3:**
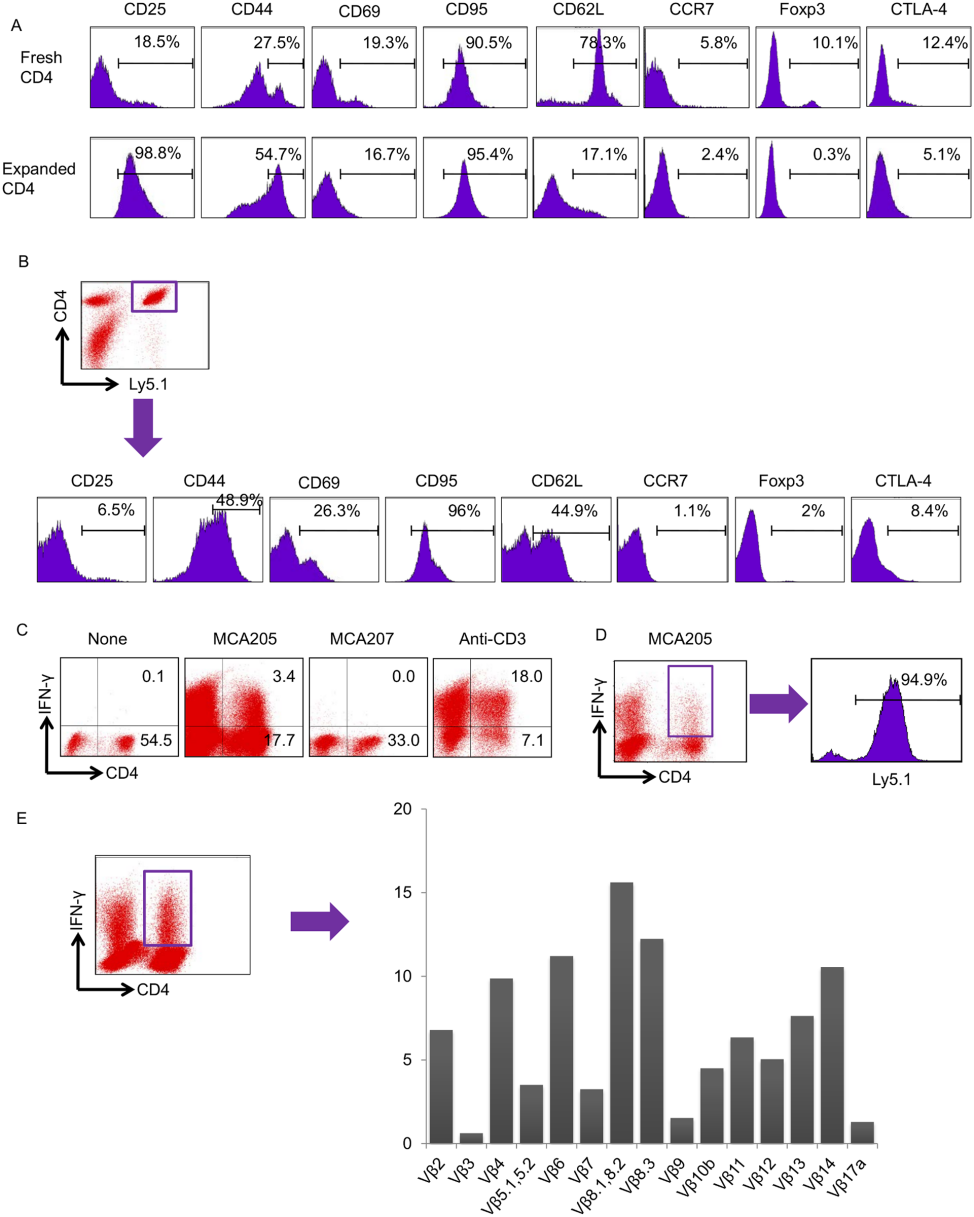
Tumor-specific effector CD4^+^ T cells were mainly induced from donor cells and maintained TCR diversity. (A) The phenotypes of freshly harvested CD4^+^ T cells and ex vivo—expanded CD4^+^ T cells were assessed by flow cytometry. A representative result from 3 independent experiments is shown. (B) Irradiated mice were transferred i.v. with ex vivo—expanded CD4^+^Ly5.1^+^ T cells and inoculated s.c. with MCA205 tumor cells. Twelve-day TDLNs were harvested and analyzed by FACS. Histograms demonstrate the phenotypes of the CD4^+^Ly5.1^+^ donor T cells. (C) Irradiated mice were reconstituted with ex vivo—expanded CD4^+^ T cells and inoculated s.c. with MCA205 tumor cells. Twelve days later, TDLN cells were activated in vitro with anti-CD3 and IL-2. These TDLN cells were tested for IFN-γ production after specific or nonspecific stimulation. (D) Irradiated mice were transferred i.v. with ex vivo—expanded CD4^+^Ly5.1^+^ T cells and were inoculated s.c. with MCA205 tumor cells. Twelve-day TDLNs were harvested and activated in vitro using anti-CD3 and IL-2. These TDLN cells were further stimulated with MCA205 tumor cells and stained for IFN-γ. Histograms show the percentage of Ly5.1^+^ donor T cells among CD4^+^ T cells responding to specific tumor antigens. (E) To examine whether these tumor-specific CD4^+^ T cells maintained the TCR Vβ repertoire, TDLN cells were harvested and activated using the CD3/IL-2 method followed by stimulation with MCA205 tumor cells. Data are from one experiment representative of three independent experiments.

The phenotypes of freshly harvested CD4^+^ T cells and ex vivo—expanded CD4^+^ T cells are depicted in Fig 3A. The upregulation of CD25 and CD44 and the down regulation of CD62L were observed after ex vivo activation. Next, we evaluated the phenotypes of ex vivo—expanded CD4 T^+^ cells that were transferred into lymphopenic tumor-bearing mice. Sublethally irradiated mice were reconstituted i.v. with 40 × 10^6^ ex vivo—expanded CD4^+^ T cells from Ly5.1 mice. These mice were then inoculated s.c. with MCA205 tumor cells to stimulate TDLNs. Twelve days later, TDLN cells were harvested and stained for FACS analyses. CD4^+^Ly5.1^+^ donor T cells from TDLNs showed higher expression of CD69 and CD62L and lower expression of CD25 and CD44 than did donor cells before transfer (Fig 3B).

To investigate whether tumor-specific effector T cells were induced by the combination treatment of irradiation and the transfer of ex vivo—expanded CD4^+^ T cells, mice were irradiated and transferred i.v. with 40 × 10^6^ ex vivo—expanded CD4^+^ T cells, followed by inoculation of MCA205 tumor cells. Twelve-day TDLNs were harvested, and the TDLN cells were activated in vitro with anti-CD3 mAbs for 2 days and then stimulated in CM containing low doses of IL-2 (16 U/ml) for 3 days, as described in our previous studies [16]. IFN-γ production from these activated TDLN cells was assessed after further stimulation with fresh MCA205 tumor digests. The tumor digests included CD11b^+^MHC-classII^+^ antigen-presenting cells (APCs), which present tumor antigens specific for CD4^+^ T cells as previously described [23]. As shown in Fig 3C, 3.4% of the TDLN cells were CD4^+^ tumor-specific T cells. Next, we assessed whether antitumor effector T cells were primed from transferred donor cells. Sublethally irradiated mice were reconstituted with ex vivo—expanded CD4^+^Ly5.1^+^ T cells, followed by the injection of MCA205 tumor cells. Twelve days later, TDLN cells were activated according to the anti-CD3/IL-2 method and then stimulated with specific MCA205 cells. FACS analysis revealed that 94.9% of the CD4^+^ T cells responding to tumor-specific stimulation were from transferred donor cells (Fig 3D). To evaluate whether these tumor-specific CD4^+^ T cells could maintain the diversity of the T cell repertoire, the expression of TCR Vβ in CD4^+^IFN-γ^+^ T cells was analyzed by FACS. As shown in Fig 3E, a broad repertoire of TCR Vβ on CD4^+^ tumor-specific T cells was observed.

### Long-lived memory T cells from ex vivo—Expanded CD4^+^ tumor-specific T cells lead to successful rejection of specific tumor re-challenge

**Fig 4:**
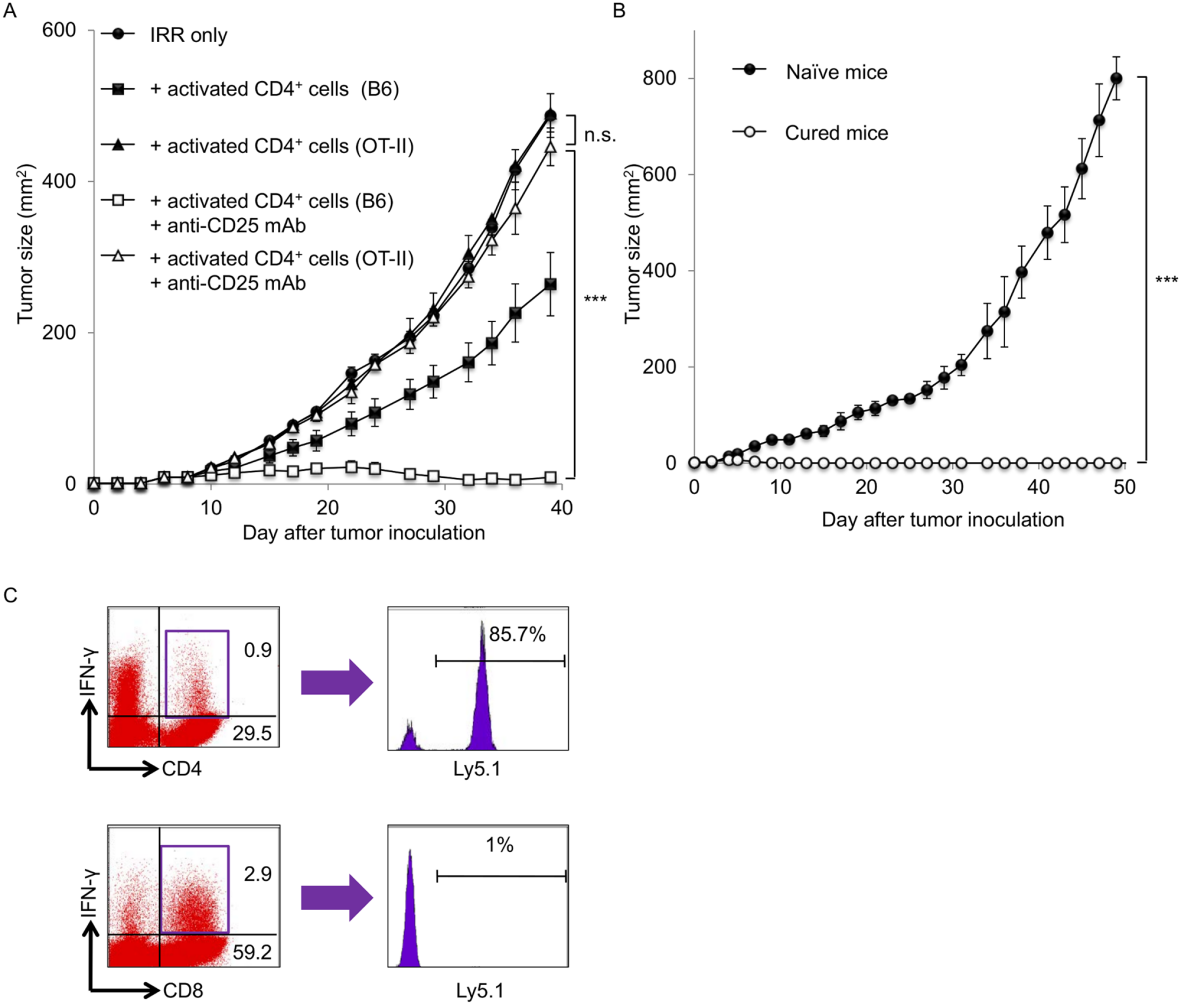
Long-term persistence of ex vivo—Expanded donor effector T cells. (A) Irradiated lymphopenic mice were reconstituted with ex vivo—expanded CD4^+^ T cells from transgenic OT-II mice or normal mice. These mice were injected with anti-CD25 mAbs following the inoculation of MCA205 tumor cells. (B) Mice were treated with combination therapy, which consisted of irradiation, the transfer of ex vivo—expanded CD4^+^ T cells and Treg depletion, and inoculated s.c. with MCA205 tumor cells. Mice that were cured of tumors were re-challenged with MCA205 tumor cells 90 days after the combination therapy. (A, B) Data are shown as mean ± SEM of 5 mice per group and are from one experiment representative of two independent experiments. ***p < 0.001; two sided Student’s *t* test. (C) Irradiated mice were transfused i.v. with ex vivo—expanded CD4^+^Ly5.1^+^ T cells and were inoculated s.c. with MCA205 tumor cells. These mice were treated with anti-CD25 mAbs. Ninety-days after successful combination therapy, spleens were harvested from cured mice. These spleen cells were activated with anti-CD3 and IL-2 followed by stimulation with MCA205 tumor cells and were tested for IFN-γ secretion in vitro. Data shown are from one experiment representative of two independent experiments.

To determine whether the ability to recognize tumor antigens is responsible for the enhancement of antitumor immunity in this model system, we transferred ex vivo—expanded CD4^+^ T cells from OT-II transgenic mice into sublethally irradiated lymphopenic mice. These mice were then inoculated s.c. with MCA205 tumor cells along the midline of the abdomen and treated with anti-CD25 mAbs to deplete Tregs. As shown in Fig 4A, the antitumor effects of the combination of irradiation, transfer of ex vivo—expanded CD4^+^ T cells from wild-type mice and Treg depletion were significantly reduced when ex vivo—expanded CD4^+^ T cells from OT-II mice were used as donor cells. Next, we examined whether long-term antitumor memory developed in mice treated with combination therapy consisting of irradiation, ex vivo—expanded T cell transfer and Treg depletion. Irradiated lymphopenic mice were reconstituted with ex vivo—expanded CD4^+^ T cells and inoculated with MCA205 tumor cells. These mice were Treg-depleted on the same day. Ninety days later, the cured mice were inoculated s.c. with 3 × 10^6^ MCA205 tumor cells, and these mice successfully rejected MCA205 tumor cells (Fig 4B). To assess whether both CD4^+^ and CD8^+^ memory T cells against MCA205 tumors were maintained for a long time, we harvested 90-day spleen cells from mice that were cured of MCA205 tumors via treatment with irradiation, transfer of ex vivo CD4^+^ T cells from Ly5.1 mice and Treg depletion. The spleen cells were activated using the CD3/IL-2 method and stimulated with MCA205 tumor cells. As shown in Fig 4C, IFN-γ production from both CD4^+^ and CD8^+^ T cells was observed after specific tumor-antigen stimulation. We further evaluated the origin of these antitumor memory T cells. FACS analysis demonstrated that the majority of the tumor-specific CD4^+^ T cells were from transferred donor T cells (85.7%). As expected, almost all tumor-specific CD8^+^ T cells originated from recipient mice (99%). These data prompted us to evaluate the role of recipient T cells in our model system. If we used Rag2^-/-^ mice as recipients, the antitumor effects of the combination of irradiation, transfer of ex vivo—expanded CD4^+^ T cells from wild-type mice and Treg depletion were significantly decreased (S1 Fig).

### DC vaccination augments the antitumor effects of combination therapy of lymphodepletion and ex vivo—Expanded CD4^+^ T cells

**Fig 5:**
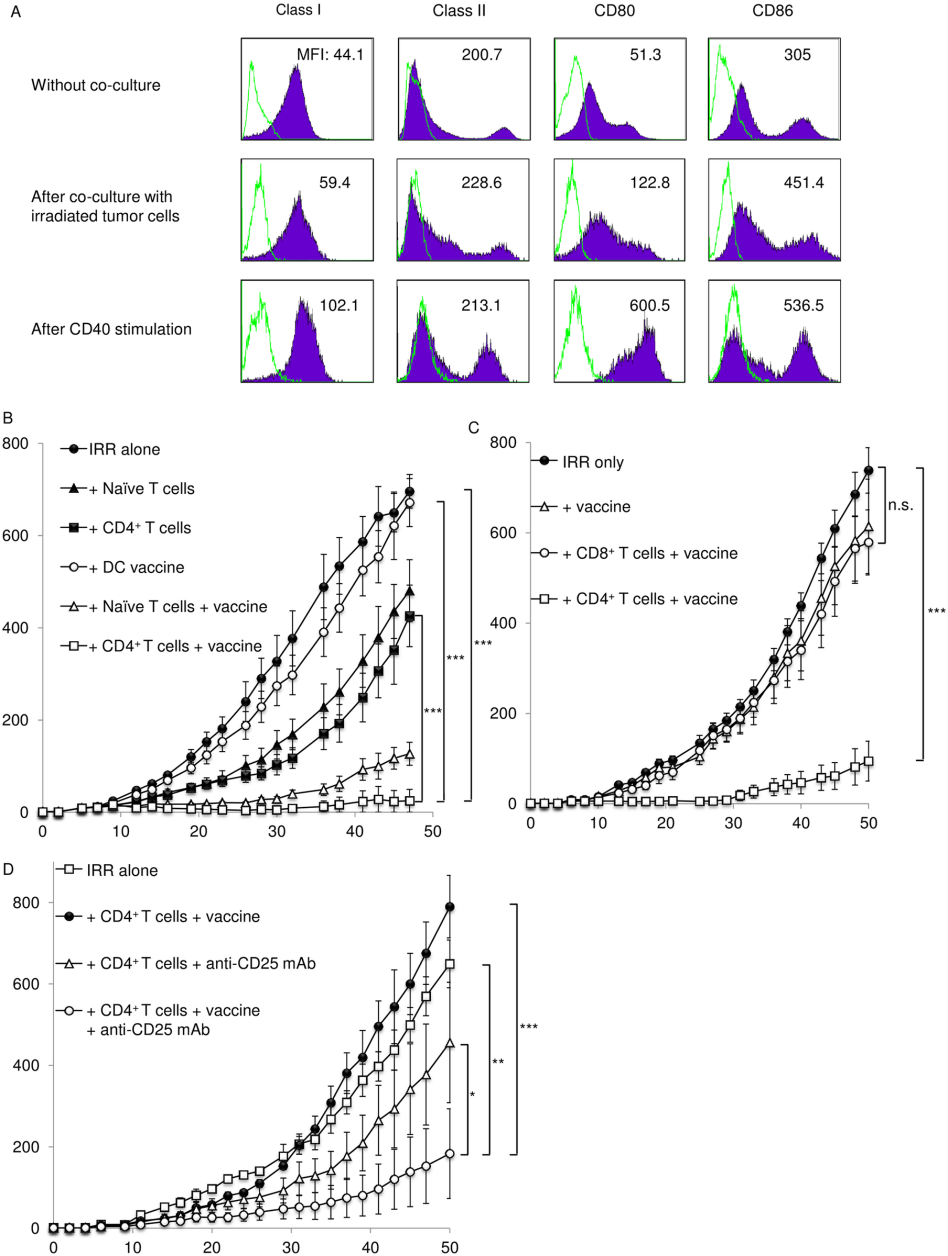
DC vaccination enhanced the antitumor effects of transfer of ex vivo—Expanded CD4^+^ T cells. (A) DCs were generated from bone marrow cells as described in the Materials and Methods section. These DCs were co-cultured with irradiated MCA205 tumor cells (50Gy) overnight and stimulated with immobilized agonistic anti-CD40 mAbs for 3 hours. Histograms show the phenotypes of CD11c^+^ DCs co-cultured with or without irradiated tumor cells, and after CD40 stimulation. Mean fluorescence intensities (MFI) of Class I, Class II, CD80 and CD86 in CD11c^+^ DCs are also indicated. Data shown are from one experiment representative of three independent experiments. (B) Irradiated mice were transferred i.v. with ex vivo—expanded CD4^+^ T cells or whole spleen cells from normal mice as the source of naïve T cells. These mice were then vaccinated with DCs and inoculated s.c. with MCA205 cells. (C) Irradiated mice were reconstituted with either ex vivo—expanded CD4^+^ or CD8^+^ T cells followed by inoculation of MCA205 tumor cells. These mice were vaccinated s.c. with DCs. (D) Mice bearing 3-day skin tumors were irradiated and transferred i.v. with ex vivo—expanded CD4^+^ T cells. These mice were then vaccinated s.c. with DCs and treated with anti-CD25 mAbs. (B, C, D) Data are shown as mean ± SEM of 5 mice per group and are from one experiment representative of two to three independent experiments. *p < 0.05, **p < 0.01, ***p < 0.001 (two sided Student’s *t* test).

Previously, we demonstrated that vaccination with DCs is capable of augmenting the priming of antitumor effector T cells in draining lymph nodes (LNs) [20]. A short duration of DC stimulation with agonistic anti-CD40 mAbs in vitro can enhance the migration of DCs to draining LNs and the ability of DCs to present tumor antigens to tumor-specific T cells. To augment the antitumor effects of the transfer of ex vivo—expanded CD4^+^ T cells and irradiation, we investigated DC vaccination in this model system. DCs were generated as described in the Materials and Methods section. For antigen loading, DCs were co-cultured with irradiated MCA205 tumor cells (50 Gy) overnight. Fig 5A demonstrates the phenotypes of DCs which were co-cultured with irradiated MCA205 tumor cells or without co-culture. These DCs co-cultured with irradiated tumor cells were then stimulated with immobilized agonistic anti-CD40 mAbs for 3 hours. Fig 5A also shows the phenotypes of DCs after stimulation with anti-CD40 mAb in vitro. The expression levels of class II, CD80 and CD86 were upregulated after CD40 stimulation. Next, we examined whether vaccination with these DCs could enhance the antitumor effects of the transfer of ex vivo—expanded CD4^+^ T cells after irradiation. Irradiated lymphopenic mice were inoculated s.c. with MCA205 tumor cells. On the same day, these mice were transferred i.v. with ex vivo—expanded CD4^+^ T cells and vaccinated with DCs that were co-cultured with MCA205 tumor cells and stimulated with anti-CD40 mAbs. Fig 5B shows that DC vaccination augmented the antitumor therapeutic responses mediated by the transfer of ex vivo—expanded CD4^+^ T cells following irradiation. By contrast, DC vaccination and the transfer of ex vivo—expanded CD8^+^ T cells did not show significant antitumor effects (Fig 5C). Next, we evaluated the combination of irradiation, the transfer of ex vivo—expanded CD4^+^ T cells, Treg depletion and DC vaccination in the 3-day skin tumor model. Mice were inoculated s.c. with 1 × 10^5^ of MCA205 tumor cells. Three days later, these mice were irradiated and transferred i.v. with ex vivo—expanded CD4^+^ T cells followed by Treg depletion and DC vaccination. As shown in Fig 5D, this combination therapy consisting of irradiation, the transfer of ex vivo—expanded CD4^+^ T cells, Treg depletion and DC vaccination significantly inhibited skin tumor growth.

## Discussion

Adoptive cell transfer (ACT) using antigen-specific effector T cells is one of the most effective immunotherapies [24, 25]. Tumor-infiltrating lymphocytes and gene-engineered T cells, such as chimeric antigen receptor—modified T cells and TCR-engineered T cells, have shown promising antitumor effects against many solid tumors and hematologic malignancies. However, there are barriers, such as the availability of tumor-infiltrating lymphocytes, on-target but off-tumor toxicities and difficulty related to the selection of suitable target antigens, that limit the wide dissemination of adoptive cell therapy. We have investigated the enhancement of antitumor immunity through the induction of tumor-specific effector T cells from naïve T cells during homeostatic proliferation [3, 4]. Our previous studies demonstrated that the combination of lymphodepletion regimens and the transfer of naïve T cells induces tumor-specific effector T cells and suppresses tumor progression. Moreover, the depletion of host Tregs that are resistant to lymphodepletion results in the successful treatment of advanced tumor models when combined with lymphodepletion by cyclophosphamide and the transfer of naïve T cells [4]. Extensive studies have revealed that tumors induce immune suppression to evade antitumor T cell responses [13–15]. Several mechanisms have been suggested for the induction of immune suppression by tumors, including induction of Tregs, myeloid-derived suppressor cells, tumor-associated macrophages and tolerogenic DCs; secretion of immunosuppressive soluble factors; and activation of negative regulatory pathways such as CTLA-4 and PD-1. This immunosuppressive tumor network interferes with the function of T cells in tumor-bearing hosts. Similarly, we previously reported that myeloid-derived suppressor cells inhibited the priming of effector T cells [19]. Furthermore, in cancer patients, down-regulation of the TCR ζ chain has been reported; this impairs T cell signaling and contributes to T cell dysfunction [11, 12]. Because the homeostatic proliferation of T cells during recovery from lymphopenia depends on signaling through the TCR and homeostatic cytokines, it appears that both effector T cells and naïve T cells are suppressed in tumor-bearing hosts [10]. Although we and others have demonstrated that the transfer of naïve T cells following lymphodepletion augments antitumor effects, the collection of fully functional naïve T cells from tumor-bearing hosts remains difficult. Thus, we sought to establish culture methods to obtain sufficient numbers of adequate naïve T cells capable of differentiating into tumor-specific effector T cells.

In the current study, we examined the ability of in vitro—stimulated naïve T cells to induce tumor-specific effector T cells and enhance antitumor immunity. Because previous studies have demonstrated that effector T cells can be efficiently expanded under specific conditions, such as supplementation with IL-2 and IL-7 for the CD8 subset or IL-7 and IL-23 for the CD4 subset after anti-CD3 stimulation, we used the same culture methods for the expansion of naïve T cells [17, 23]. This in vitro stimulation resulted in the hyperexpansion of both CD4^+^ and CD8^+^ naïve T cells (Fig 1A and 1B). FACS analyses revealed that the TCR diversity was maintained after ex vivo expansion (Fig 1C). The transfer of these polyclonal ex vivo—expanded T cells into lymphopenic hosts significantly inhibited tumor progression (Fig 2A). Similar to our findings, Dummer et al. reported that the transfer of polyclonal naïve T cells into lymphopenic mice significantly enhanced antitumor immunity [2]. Recent evidence supports the association of the antitumor effects of immunotherapy and a number of neoantigens [26–28]. PD-L1/PD-1 blockade therapy is more efficacious toward cancer cells, which have a higher neoantigen burden [27, 28]. PD-L1/PD-1 blockade therapy recovers the function of exhausted tumor-specific effector T cells and shows antitumor effects. Thus, tumor cells with a large number of neoantigens seem to strongly induce tumor-specific T cells that have been suppressed through immune checkpoints. Indeed, the density of tumor-infiltrating lymphocytes has been reported to be correlated with the response to PD-L1/PD-1 blockade therapy [29, 30]. These findings indicate that the induction of tumor-specific effector T cells may augment the effectiveness of immune checkpoint inhibitors. We are currently investigating whether the transfer of ex vivo—expanded T cells enhances the antitumor effects of PD-L1/PD-1 blockade therapy, and our results show that the transfer of ex vivo—expanded T cells into mice treated with lymphodepletive therapy successfully augmented the efficacy of anti-PD-1 treatment (unpublished data).

Previous studies investigating adoptive cell transfer have mainly focused on the role of CD8^+^ T cells [24, 31]. Adoptive transfer of activated tumor-specific CD8^+^ T cell has demonstrated durable antitumor effects. Recent evidence has also demonstrated that the transfer of CD4^+^ effector T cells augments antitumor immunity and inhibits tumor progression [32, 33]. Tumor-specific CD4^+^ T cells acquire cytotoxic functions and eradicate tumor cells in vivo when these cells are transferred into lymphopenic hosts [34, 35]. Moreover, our previous studies have shown that the transfer of naïve CD4^+^ T cells into lymphodepleted hosts augments antitumor immunity [3, 4]. In the current study, the transfer of ex vivo—expanded CD4^+^ T cells significantly delayed skin tumor growth (Fig 2B). By contrast, the transfer of ex vivo—expanded CD8^+^ T cells showed minimal antitumor effects (Fig 2B). We have previously demonstrated that the percentage of Tregs increases after lymphodepletion [3, 4], and depletion of Tregs that survived lymphodepletion augmented the antitumor effects of the transfer of naïve T cells. Consistent with these studies, the depletion of Tregs after irradiation significantly inhibited skin tumor growth in mice reconstituted with ex vivo—expanded CD4^+^ T cells (Fig 2C). Again, the combination of Treg depletion and transfer of ex vivo—expanded CD8^+^ T cells failed to show strong antitumor effects (Fig 2D). Antitumor effector CD4^+^ T cells that respond to specific tumor-antigen stimulation were primed in TDLNs, and the majority of these tumor-specific CD4^+^ T cells were induced from donor CD4^+^ T cells (Fig 3C and 3D). Furthermore, tumor-specific CD4^+^ T cells maintained TCR diversity (Fig 3D). These findings indicate that ex vivo—expanded CD4^+^ T cells that recognize tumor antigens mediate the augmentation of antitumor immunity. Indeed, the transfer of ex vivo—expanded CD4^+^ T cells from OT-II transgenic mice completely abolished the antitumor effect observed in this model system (Fig 4A).

One of the major goals of tumor immunotherapy is to induce antitumor memory T cells that survive for long periods to prevent tumor recurrence. In this study, we demonstrated that mice cured of tumors by combination therapy, consisting of the transfer of ex vivo—expanded CD4^+^ T cells after irradiation and Treg depletion, successfully rejected the challenge of MCA205 tumor cells 90 days after the first inoculation of MCA205 cells (Fig 4B). We further showed that tumor-specific CD4^+^ and CD8^+^ T cells were present in the spleens of cured mice 90 days after the administration of combination therapy (Fig 4C). The majority of long-lived tumor-specific CD4^+^ T cells originated from ex vivo—expanded donor cells, whereas tumor-specific CD8^+^ T cells were primed from irradiated recipient cells (Fig 4C).

In this study, CD4^+^ T cells were responsible for the augmented antitumor immunity (Fig 2B). MCA205 tumor cells do not express MHC class II, and CD4^+^ T cells are not able to directly recognize MCA205 tumor cells, suggesting that APCs are involved in this augmentation. Although DC-based vaccines have been thoroughly investigated in clinical trials, very few DC vaccines have demonstrated objective responses [36, 37]. Anti-tumor DC vaccines are generally designed to activate the function of effectors, including tumor-specific T cells. The current study found that lymphodepletion and the transfer of ex vivo—expanded CD4^+^ T cells could induce polyclonal tumor-specific T cells (Fig 3C and 3E). These findings prompted us to examine whether DC vaccines could enhance the antitumor immune responses underlying the transfer of ex vivo CD4^+^ T cells and lymphodepletion. We previously described that a short duration of CD40 stimulation augmented the migration activity of DCs and efficiently induced antitumor effector T cells [20]. In the current study, DCs were stimulated with agonistic anti-CD40 mAbs after tumor-antigen loading and were inoculated s.c. into mice that were irradiated and reconstituted with ex vivo—expanded CD4^+^ T cells. DC vaccination combined with the transfer of CD4^+^ T cells and lymphodepletion significantly inhibited tumor progression (Fig 5B).

Lymphocytes from cancer patients consist of several types of T cells, such as effector T cells and naïve T cells. Previous studies have focused on antitumor effector T cells that were enriched and stimulated in vitro for adoptive cell therapy. The current findings indicate that naïve T cells show antitumor effects after ex vivo expansion under specific conditions.

## Supporting information

S1 Fig The antitumor effects of combination therapy of lymphodepletion, transfer of ex-vivo expanded CD4^+^ T cells and Treg depletion required recipient cells. Rag2^-/-^ mice were irradiated and reconstituted with ex vivo-expanded CD4^+^ T cells and were inoculated s.c. with MCA205 tumor cells. These mice were treated with anti-CD25 mAbs. Data are shown as mean ± SEM of 5 mice per group and are from one experiment representative of two independent experiments. ***p < 0.001; two sided Student’s *t* test.
(TIF)
