# Supplementary figures and images for: Transfer of in vitro-expanded naïve T cells after lymphodepletion enhances antitumor immunity through the induction of polyclonal antitumor effector T cells
Source: PLoS One. 2017 Aug 30;12(8):e0183976. doi: 10.1371/journal.pone.0183976 (PMC5576657; doi:10.1371/journal.pone.0183976)

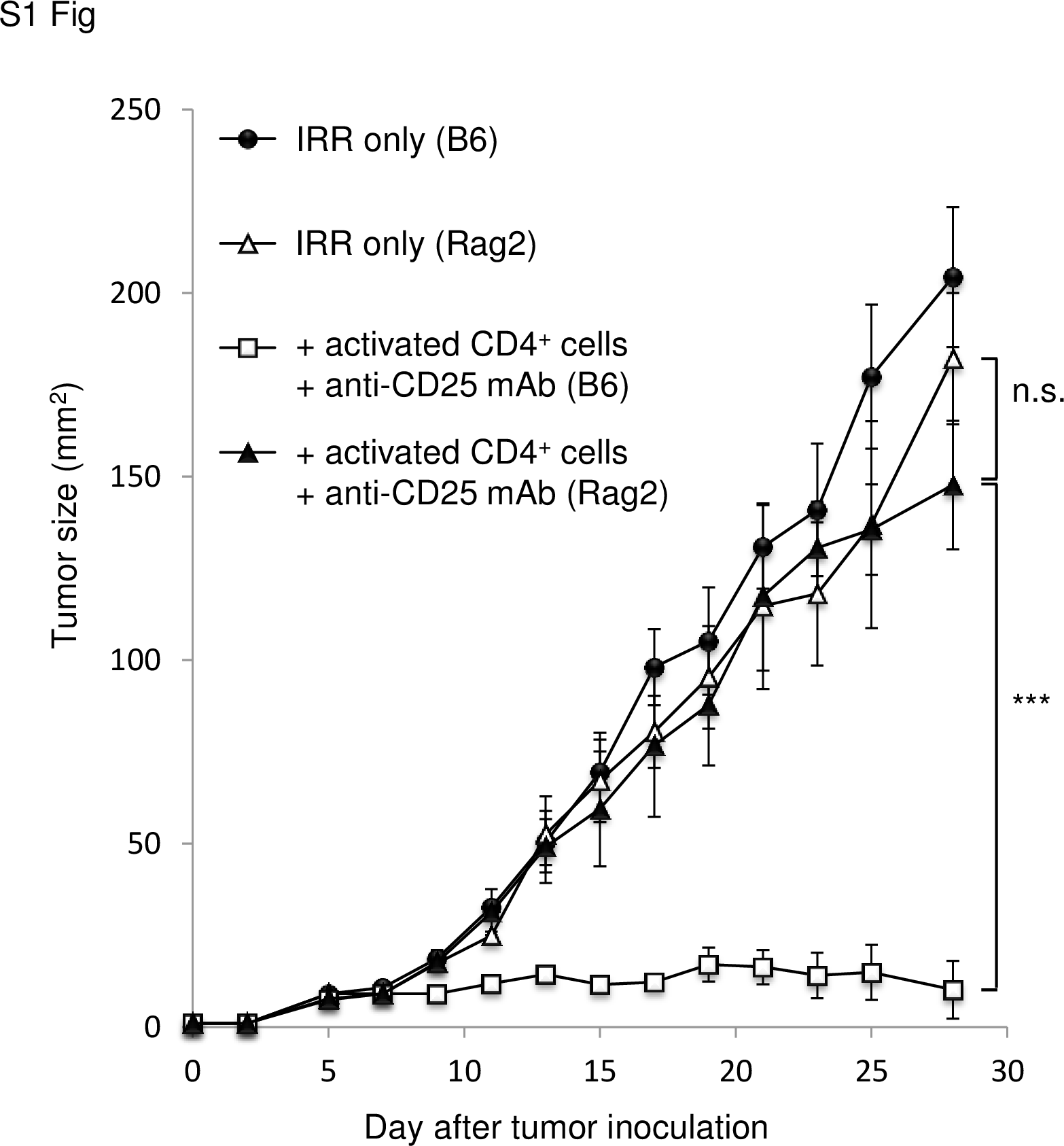

Supplement: S1 Fig — Rag2-/- mice were irradiated and reconstituted with ex vivo-expanded CD4+ T cells and were inoculated s.c. with MCA205 tumor cells. These mice were treated with anti-CD25 mAbs. Data are shown as mean ± SEM of 5 mice per group and are from one experiment representative of two independent experiments. ***p < 0.001; two sided Student’s t test. (TIF) [file pone.0183976.s001.tif]
